# Bilateral Versus Single Internal Thoracic Artery Grafts

**DOI:** 10.1007/s11886-018-0947-1

**Published:** 2018-01-23

**Authors:** Michael Persson, Ulrik Sartipy

**Affiliations:** 1 Department of Molecular Medicine and Surgery, Karolinska Institutet, Stockholm, Sweden; 2 Section of Cardiothoracic Surgery, Heart and Vascular Theme, Karolinska University Hospital, SE-171 76 Stockholm, Sweden

**Keywords:** CABG, Arterial grafting, Internal thoracic artery, BITA, Long-term survival, Sternal wound infections

## Abstract

**Purpose of Review:**

Several advances have been made in recent years to improve outcome for patients with coronary artery disease. One of the most debated topics regarding surgical treatment with coronary artery bypass grafting (CABG) is graft selection. This review aims to present the current status and scientific evidence for bilateral internal thoracic artery (BITA) grafting.

**Recent Findings:**

Observational studies and pooled analyses suggest that BITA grafting is associated with improved survival. Early results from a large randomized controlled trial report safety and efficacy of the method. The improved survival might be amplified in select groups, but with an increase in sternal wound-related complications. The benefit of BITA grafts seems to remain to an approximate age of 69 years at surgery.

**Summary:**

CABG with BITA grafts is likely associated with improved long-term survival at a cost of an increase in sternal wound infections. Ten-year results from the Arterial Revascularization Trial are expected in 2018, providing the best evidence regarding the method yet. Early results show it is a safe method in most patient categories considerable for CABG.

## Introduction

The history of coronary artery bypass grafting (CABG) goes back to the 1960s, when Dr. Goetz is said to have performed the first procedure [1]. As the incidence of coronary artery disease increased during the twentieth century, the procedure became more popular and widespread as the best treatment for the condition. The use of CABG for the treatment of symptomatic coronary artery disease has declined over the early twenty-first century, primarily due to advances in percutaneous coronary intervention (PCI). In European guidelines on myocardial revascularization, CABG has a class I recommendation for all types of stenosis location except for “one or two-vessel disease without proximal LAD stenosis” [2]. Thus, it remains the superior method for the treatment of selected patient groups.

Over the decades, advances in perioperative, postoperative, and surgical technique have improved outcomes for patients with coronary artery disease. One of the milestones in this progress was the discovery of the left internal thoracic artery (LITA) as a superior graft vessel [3]. To this day, LITA to LAD grafting remains the gold standard of the procedure [4]. This finding led to the idea that arterial graft vessels are probably better suited as arterial conduits than their venous counter-parts such as the saphenous vein graft (SVG). The reason for the superiority of the LITA over the SVG can be explained by differences in long-term patency. Numerous trials have reported a 10-year patency of the LITA of 90–95% compared to 50% in SVG [5, 6]. Hence, the use of SVG in a patient with a life expectancy exceeding 10 years increases their risk of the need for repeat revascularization. Therefore, various graft vessels have been tried as the second conduit to complement the LITA to LAD graft.

One early suggested alternative is the radial artery as proposed by Carpentier et al. in 1973 [7, 8]. The potential benefit is that it is an easily harvested arterial conduit. The major draw-back is its tendency for spasm, acknowledged even by the group first to recommend its use [9, 10]. Even though this can be managed with anti-spasmodic drugs, it theoretically increases the risk for peri- and postoperative complications.

Another possible graft vessel is the gastroepiploic artery. A potential risk with this vessel is that it requires a laparotomy for access; however, it has been reported to be safe to use with no increase in wound infections, gastric ischemia, or overall complications. Ten-year patency has been reported to be 70% [11].

The differences in patency rates between arterial conduits reflects the fact they are different in terms of histological and biological features, and may thus pose different advantages and disadvantages as graft vessels in CABG.

Important histological features of the ITA include an elastic intimal lamina, paucity in intimal fenestration, relatively sparse media, and sparse *vasa vasorum* in the adventitia [12, 13]. In comparison, the radial artery has a relatively thick media and a tendency for distal intimal hyperplasia [9]. Further differences can be observed with the SVG. They have sparser elastic intimal lamina and sequential thickening composed of layered structures of smooth muscle cells and collagen combined with focal absence of collagen. The hemodynamic stress exerted by arterial pressure on the venous wall results in intimal thickening and adventitial to intimal migration of the *vasa vasorum.* Over time, these factors contribute to endothelial damage, inflammation, foam-cell formation, atherosclerosis, and plaque rupture [13].

Even though the radial artery is an arterial vessel, it still seems more susceptible to atherosclerosis than the ITA. Rates of atherosclerosis at harvest has been reported to be 5.3% in the radial artery, compared to 0.7% in the ITA [14].

The advantages of the LITA seems reasonable to extrapolate to the RITA. A study by Tatoulis et al. reported similar patency rates in LITA and RITA grafts, 96.5 and 94.6% respectively [15]. One of the hallmark studies on the usage of BITA was published in 1999 by Lytle and colleagues. This retrospective cohort consisted of 10,124 patients (SITA 8123, BITA 2001) who underwent surgery between 1971 and 1989. They reported an improved long-term survival and reduced need for repeat revascularization in the BITA group [16]. These results were further supported by a meta-analysis by Taggart and colleagues in 2001 [17]. Their analysis included seven studies with a total of 15,962 patients (SITA 11269, BITA 4693). The overall finding was a significantly improved survival in the BITA group (HR 0.81, 95% CI 0.70–0-94) [17]. Despite these findings, there seems to be a reluctance in the surgical community to adopt the method. In a nationwide US study, the rate of BITA use was reported to be 3.9% [18]. This is comparable to 1% in Sweden and 10% in the UK and Ireland [19, 20]. The main reason for not using the RITA is likely out of fear for sternal wound complications [21].

Not all studies have reported benefits with the use of BITA grafting. A nationwide observational cohort by Dalén and colleagues included 49,702 patients with a mean follow-up of 7.5 years [19]. After adjustment, there was no difference in survival rate between SITA and BITA (HR 1.16, 95% CI 0.97–1.37) [19].

Table 1 summarizes selected studies on BITA grafting and its effect on long-term survival. Although the majority of studies demonstrate beneficial effects of BITA grafts, it is noteworthy that one of the larger studies performed by Dalén and colleagues failed to do so [19].

**Table 1:** Long-term survival, BITA vs. SITA

Author	Year of publication	Operation years	No. of patients	10-year survival SITA	10-year survival BITA	HR (95% CI)
Buttar et al. [45••]	2017	1972–2013	89,399	NA	NA	0.78 (0.72–0.84)
Benedetto et al. [46]	2014	2001–2013	4195	89.8%	93.9%	0.61 (0.38–0.97)
Dalén et al. [19]	2014	1997–2008	49,702	76%	71%	1.04 (0.78–1.4)
Dorman et al. [39]	2012	1972–1994	1107	~ 50%	~ 65%	0.77 (0.64–0.91)
Locker et al. [47]	2012	1993–2009	8622	80%	83%	0.79 (0.66–0.94)
Taggart et al. [17]	2001	1971–1995	15,962	NA	NA	0.81 (0.70–0.94)
Lytle et al. [16]	1999	1971–1989	10,124	79%	84%	0.60 (0.53–0.68)

*SITA* single internal thoracic artery, *BITA* bilateral internal thoracic artery, *HR* hazard ratio, *CI* confidence interval

A selection of studies regarding the effect of BITA grafts on sternal wound infections (SWI) are summarized in Table 2. Here, the majority of studies demonstrate a statistically significant risk increase for SWI. However the largest study by Itagaki and colleagues did not find an overall risk increase for BITA patients, except in severely diabetic patients as discussed below [18].

**Table 2:** Sternal wound infection, BITA vs. SITA

Author	Year of publication	Operation years	No. of patients	SITA	BITA	OR (95% CI)
Buttar et al. [45••]	2017	1972–2013	89,399	0.9%	1.6%	1.59 (1.06–2.40)*
Benedetto et al. [46]	2014	2001–2013	4195	1.5%	2.1%	NA
Dalén et al. [19]	2014	1997–2008	49,702	NA	NA	1.71 (1.01–2.88)
Dai et al. [48]	2013	1984–2010	172,880	1.6%	2%	1.61 (1.41–1.82)**
Itagaki et al. [18]	2013	2002–2008	1,526,360	1.4%	1.3%	1.03 (0.96–1.10)
Dorman et al. [39]	2012	1972–1994	1107	1.7%	3.1%	NA
Puskas et al. [49]	2012	2002–2010	3527	1.0%	1.2%	1.85 (0.79–4.35)
Taggart et al. [22]	2010	2004–2007	3102	0.6%	1.9%	3.24 (1.54–6.83)*
Lytle et al. [16]	1999	1971–1989	10,124	1.4%	2.5%	NA

*SITA* single internal thoracic artery, *BITA* bilateral internal thoracic artery, *OR* odds ratio, *CI* confidence interval, *NA* not available

*Only including PS-adjusted studies

**Relative risk

To address the question of potential benefits and risks associated with BITA grafting, Taggart and colleagues initiated the Arterial Revascularization Trial (ART). At the 1-year interim report, early mortality and major adverse cardiac clinical events were low and similar in both groups. However, the BITA group reported a 1.3% absolute increase in the need for sternal wound reconstruction [22].

Current guidelines regarding BITA grafting reflects the lack of conclusive evidence for the method. In the American Society of Thoracic Surgeons guidelines on arterial revascularization, this is expressed as “Use of bilateral ITAs should be considered in patients who do not have an excessive risk of sternal complications” (class IIa, level B) [23]. Similar recommendations are made in European guidelines on myocardial revascularization, “Bilateral mammary artery grafting should be considered” (class IIa, level B) [2]. As the community awaits the final results of the ART trial, surgeons have to rely on observational and pooled-data analysis to make informed decisions regarding graft selection, patient selection and risk-balance considerations. This review aims to provide a short overview of the current status and evidence for BITA grafting.

## The ART Trial

The only randomized trial of bilateral versus single internal thoracic grafts to date is the Arterial Revascularization Trial by Taggart and colleagues [22, 24••]. The trial included patients from 28 centers in seven different countries from June 2004 to December 2007. The primary outcome measure is a comparison of all-cause mortality at 10-year follow-up. Sample size calculations showed that 2928 patients were needed to detect an absolute difference of 5% in the primary outcome measure with 90% power and a 5% significance level. 3102 patients were enrolled. 1548 patients were assigned to the BITA group and 1294 underwent BITA-CABG [22, 24••]. Concerns have been raised regarding that a large number of patients randomized to BITA received SITA grafts, and to a lesser extent, were lost to follow-up. Consequently, this could decrease the power of the study so that it no longer will be able to detect a difference at 10-year follow-up [25]. At the 1-year interim report, 30-day mortality was 1.2% in both groups and the rate of MACCE were < 2%. Sternal wound reconstruction frequency was 1.9 vs. 0.6% for BITA and SITA, respectively (RR 3.24, 95% CI 1.54–6.83) [22]. At 5-year follow-up, the rate of death was 8.7 and 8.4% for BITA and SITA, respectively (HR 1.04, 95% CI 0.81–1.32) [24••].

## Harvest Technique

One of the most common approaches to avoid sternal wound complications after BITA harvest is modification of the harvest technique. The two most common techniques practiced today is the pedicled harvest and the skeletonized harvest. In a pedicled harvest, the substernal fascia, surrounding veins and adipose tissue are dissected *en bloc* with the artery. In skeletonized harvest, a meticulous dissection is performed to only expose and mobilize the internal thoracic artery while trying to preserve surrounding tissue as much as possible. The harvest might be performed using sharp dissection with micro-scissors, scalpel, and limited use of low (or un-)powered electrocautery, or by the use of the harmonic scalpel. To date, there is a paucity of evidence for the use of the harmonic scalpel, but the rationale behind its use is that it is likely to cause less endothelial damage compared to electrocautery, and that it is faster than harvest without electrocautery [26, 27].

It has been suggested that the pedicled technique results in an increase in sternal wound complications due to sternal devascularization [28, 29]. Thus, the limited tissue-defect and preserved micro-circulation after skeletonized harvest might be a protective factor against SWI. This was studied in a post hoc analysis of the ART trail examining the effect of harvest technique. The researchers found that the use of the skeletonized technique in BITA harvest yielded similar rates of SWI as pedicled SITA harvest. However, the use of skeletonized harvest on SITA grafts did not result in fewer SWIs [30].

In a meta-analysis of skeletonized versus pedicled technique in diabetic patients, the results were similar. The study included ten observational studies with a total of 126,235 patients (122,465 LITA, 3770 BITA). Skeletonized BITA harvest had similar rates of SWI as pedicled LITA (overall RR 0.94 [0.42–2.09]) whereas pedicled BITA harvest was associated of an increased risk of SWI compared to LITA (overall RR 1.82 [1.42–2.33]) [31].

A meta-analysis including 22 studies and a total of 4817 patients (2424 skeletonized, 2393 pedicled) reported ORs for SWI between skeletonized and pedicled groups. The overall OR demonstrated a significant difference in favor of skeletonized technique, with consistent benefits across diabetic, BITA, and diabetic-BITA subgroups [32].

## BITA Grafting in Diabetic Patients

CABG is known to be associated with improved outcomes in patients with symptomatic multivessel CAD compared to PCI [33–36]. This benefit has been shown to be further improved by the use of arterial graft vessels [37]. The main concern over BITA grafting is likely fear of SWI, and considering that diabetes is a known risk factor for SWI, the use of BITA grafting in this patient category has been ambiguous [38].

In a 30-year follow-up of 1107 diabetic patients and 414 propensity score-matched pairs by Dorman and colleagues, BITA grafting was associated with a significantly enhanced long-term survival after a mean follow-up of 9.9 years. The median survival for SITA was 9.8 years (95% CI 8.6–10.5) and BITA, 13.1 years (95% CI 12.2–13.9, *P* = 0.003) in the matched cohort [39].

In a recent study, Raza and colleagues compared BITA to SITA + radial artery grafts and effects on SWI and late mortality. 1325 patients (SITA + RA 965, BITA 360) were followed for a median follow-up of 7.4 years. In the propensity score-matched cohort, the 14-year survival was 58 vs. 64% (*P* = 0.2) for SITA + radial artery and BITA, respectively [40]. This supports the idea that radial artery is a reasonable alternative to RITA in diabetic patients in regards of survival. In the 1-year results of the ART trial, 24% of the study population had diabetes. Of those requiring sternal wound reconstruction, 50% were diabetic [22]. In the Dorman study, there was no difference in SWI frequency (SITA 1.7%, BITA 3.1%, *P* = 0.179) [39]. This is partly in contrast to a study from The Society of Thoracic Surgeons Database including 120,793 patients with diabetes (SITA 119061, BITA 1732) where BITA grafting was associated with an OR of 2.23 for SWI (95% CI, 1.69–2.96) and no difference in short-term mortality [38]. In the study by Raza et al., the propensity score-matched cohort displayed a frequency of SWI of 1.4% in both groups [40]. Although the radial artery is a reasonable alternative to RITA in diabetic patients in regards of survival, it does not demonstrate a beneficial effect on SWI.

## BITA Grafting in Obese Patients

Similar to diabetic patients, obese patients are likely to benefit from the increased patency and resistance to atherosclerosis offered by BITA, but at a possible cost of a higher risk of SWI. Evidence regarding obesity as a risk factor for SWI has been conflicting.

In a study by Benedetto and colleagues examining BITA grafting in obese patients, a total of 1522 obese patients generated 229 propensity score-matched pairs to compare BITA vs. SITA. BITA was associated with significantly improved long-term survival after a median follow-up of 4.5 years (HR 0.35, 95% CI 0.13–0.97) and a lower rate of repeat revascularization (HR 0.45, 95% CI 0.23–0.85) [41]. In a similar retrospective study by Ruka and colleagues, 5583 obese patients were included. After propensity score matching, BITA was not associated with significantly improved long-term survival at a median follow-up of 7.4 years [42]. In a large trial of 1,526,360 patients, obesity was identified as an independent risk factor for SWI, but in the BITA group, only patients with chronic complications of diabetes had a significantly higher risk with an OR of 1.90 (95% CI 1.51–2.41) [18]. In the Benedetto cohort, the frequency of deep sternal wound infection (DSWI) was 2.6% in the BITA group and 0.9% in the SITA group, but the difference did not reach statistical significance [41]. In the Ruka study, the frequency of DSWI was 3.2% in the BITA group, and 1.1 in the SITA group (*P* < 0.001) after matching [42].

## BITA Grafting in Elderly Patients

The major reported benefit of BITA grafts is improved long-term survival. This suggests that the method might not be associated with improved outcomes in elderly patients as expected survival might be shorter than timing of expected gain.

A study by Kieser and colleagues on a cohort of 5601 patients with a mean follow-up of 7.1 years performed a spline analysis that demonstrated an intersection of risk for mortality for BITA compared to SITA at 70 years of age [43]. This suggests the benefit of BITA grafts remains up to an age of approximately 70 years. These results are supported by Benedetto and colleagues in a study of 4190 patients and a mean follow-up of 4.9 years. In their spline analysis, the intersection occurred at 69 years of age. In patients 69 years and younger, BITA was associated with significantly improved survival (HR 0.49, 95% CI 0.24–0.98) [44•].

## Conclusions

BITA grafts are still used at a low rate. Observational studies and pooled analyses suggest that use of BITA grafts is associated with improved long-term survival at a cost of an increase in sternal wound complications. Early results from the ART trial show that it is a safe method for most patient categories considerable for CABG when appropriate care is taken to minimize the risk for sternal wound complications. Special consideration is needed in patients of more than 70 years of age, and those with a high risk of sternal wound complications. Until final results from the ART trial are published, the careful recommendations for BITA use in European and American guidelines reflects the current scientific support for the method.
